# CD16 is indispensable for antibody-dependent cellular cytotoxicity by human monocytes

**DOI:** 10.1038/srep34310

**Published:** 2016-09-27

**Authors:** Wei Hseun Yeap, Kok Loon Wong, Noriko Shimasaki, Esmeralda Chi Yuan Teo, Jeffrey Kim Siang Quek, Hao Xiang Yong, Colin Phipps Diong, Antonio Bertoletti, Yeh Ching Linn, Siew Cheng Wong

**Affiliations:** 1 Singapore Immunology Network (SIgN), Agency for Science, Technology and Research (ASTAR), 8A Biomedical Grove, #04-06, Immunos, Singapore 138648, Singapore; 2 Department of Pediatrics, National University of Singapore, Centre for Translational Medicine, 14 Medical Drive Singapore 117599, Singapore; 3 Department of Haematology, Singapore General Hospital, Outram Road, Singapore 169608, Singapore; 4 Singapore Institute for Clinical Sciences, Brenner Centre for Molecular Medicine, 30 Medical Drive, Singapore 117609, Singapore; 5 Duke-NUS Medical School, 8 College Road, Singapore 169857, Singapore

## Abstract

Antibody-dependent cellular cytotoxicity (ADCC) is exerted by immune cells expressing surface Fcγ receptors (FcγRs) against cells coated with antibody, such as virus-infected or transformed cells. CD16, the FcγRIIIA, is essential for ADCC by NK cells, and is also expressed by a subset of human blood monocytes. We found that human CD16− expressing monocytes have a broad spectrum of ADCC capacities and can kill cancer cell lines, primary leukemic cells and hepatitis B virus-infected cells in the presence of specific antibodies. Engagement of CD16 on monocytes by antibody bound to target cells activated β2-integrins and induced TNFα secretion. In turn, this induced TNFR expression on the target cells, making them susceptible to TNFα-mediated cell death. Treatment with TLR agonists, DAMPs or cytokines, such as IFNγ, further enhanced ADCC. Monocytes lacking CD16 did not exert ADCC but acquired this property after CD16 expression was induced by either cytokine stimulation or transient transfection. Notably, CD16+ monocytes from patients with leukemia also exerted potent ADCC. Hence, CD16+ monocytes are important effectors of ADCC, suggesting further developments of this property in the context of cellular therapies for cancer and infectious diseases.

Immunoglobulin G (IgG) antibody subclasses play major roles in the control of bacterial and viral infections, the killing of tumour cells during antibody therapy and the pathological destruction of healthy tissues in autoimmune diseases^1^,^2^,^3^. As a result of their potency and range of actions, antibodies have become one of the most rapidly growing classes of human therapeutics in recent years, particularly in cancer treatments.

Antibodies mediate their anti-tumour effects directly, by interfering with tumor cell growth, or indirectly by activating immune-mediated complement-dependent cytotoxicity (CDC) or antibody-dependent cellular cytotoxicity (ADCC). A growing body of evidence suggests that ADCC may be the dominant mechanism operating *in vivo*^1^,^2^,^3^,^4^,^5^. The process of ADCC begins with recognition of an antigen expressed on the target cell surface by specific immunoglobulins. The Fc domain of these antibodies is then bound by Fcγ receptors (FcγRs) expressed on immune effector cells, which triggers the release of cytotoxic granules towards the target cell or upregulates death receptors expression on the cell surface. In murine cancer models, both rituximab and trastuzumab efficacy has been shown to completely depend on activating FcγRs^6^,^7^, in particular FcγRI and/or FcγRIV^8^,^9^. This appears to be similar in humans where polymorphisms in either FcγRIIa or FcγRIII that affect their affinity for IgG influence the clinical success of rituximab^10^,^11^, trastuzumab^1^,^2^,^3^,^12^,^13^,^14^ and cetuximab^4^,^5^,^6^ treatment for B-cell lymphoma, breast cancer and colorectal cancer, respectively.

NK cells are considered to be the main mediators of ADCC both in physiological and therapeutic settings. However, NK cells are present only in low numbers in the microenvironment of colorectal^7^,^8^, renal^9^,^10^, liver, skin, breast and lung carcinomas^11^,^12^,^13^,^14^. Defects in their cytotoxic function due to changes in activating and inhibiting receptor expression, upregulation in MHC class I expression, decreased expression in the signal transducing ζ chain, CD16 and cytotoxic machinery has been reported in numerous studies^15^,^16^,^17^. A recent study further showed that cross-linking of CD16 on NK cells promoted a phenomenon known as NK cell abnormalities (NKCA), which not only included CD16 down-regulation, but also an increased in the frequency of apoptotic NK cells as well as enhanced depletion of NK cells in the presence of leukemic cells^18^. Apparently, this NKCA can be overcome by inhibiting MMP activation with TIMP3^18^. Notably, trastuzumab treatment was more effective in mice lacking the inhibitory FcγRIIb^7^, which is not expressed by NK cells, implying the involvement of other immune cell populations. Using target cell depletion approaches in mice, several studies have demonstrated that monocytes and macrophages to be the principal mediators of ADCC against α-CD20-coated B cells *in vivo*^4^,^19^.

In both mice and humans, subsets of blood monocytes exhibit differential surface expression of the various FcγRs^20^. FcγRIIIA (CD16) distinguishes human monocytes into two major subsets (i.e. CD16+ and CD16−) and they can be further subdivided using additional surface markers such as CD56^21^,^22^. The minor subset that expresses low level of CD56 is mostly CD16− and is expanded in numbers under inflammatory conditions like Crohn’s disease and rheumatoid arthritis^23^,^24^. While both the CD16+ and CD16− monocyte populations express similar levels of FcγRIIA (CD32), FcγRI (CD64) is preferentially expressed on the CD16− subset^20^. The infiltration of monocytes into tumours has been widely observed. Interestingly, a recent study showed that tumour infiltration of CD16+ myeloid was associated with improved survival of colorectal cancer patients^8^. Whether CD16 expression on monocytes could promote cytotoxicity like it does in NK cells is unknown. In this study, we determine the capacity of human monocyte subsets to perform ADCC, and specifically assessed the role of CD16 in this function.

## Results

### CD16+ and not CD16− monocytes exert ADCC against antibody-coated target cells

CD16 expression distinguishes two subsets of monocytes. We determined their relative capacity to exert ADCC using antibody-coated cancer or viral infected cell lines as targets. Cancer cell lines assessed were A549 lung adenocarcinoma, Raji Burkitt’s lymphoma and SKBR3 breast adenocarcinoma because chimeric or humanized antibodies that recognize specific antigens (ganglioside GM2 on A549, CD20 on Raji and HER2 on SKBR3) on their surface are either available or already in clinical use. We first confirmed the expression of the specific antigens on each cell line by flow cytometry (Supplementary Figure 1A).

**Figure 1:**
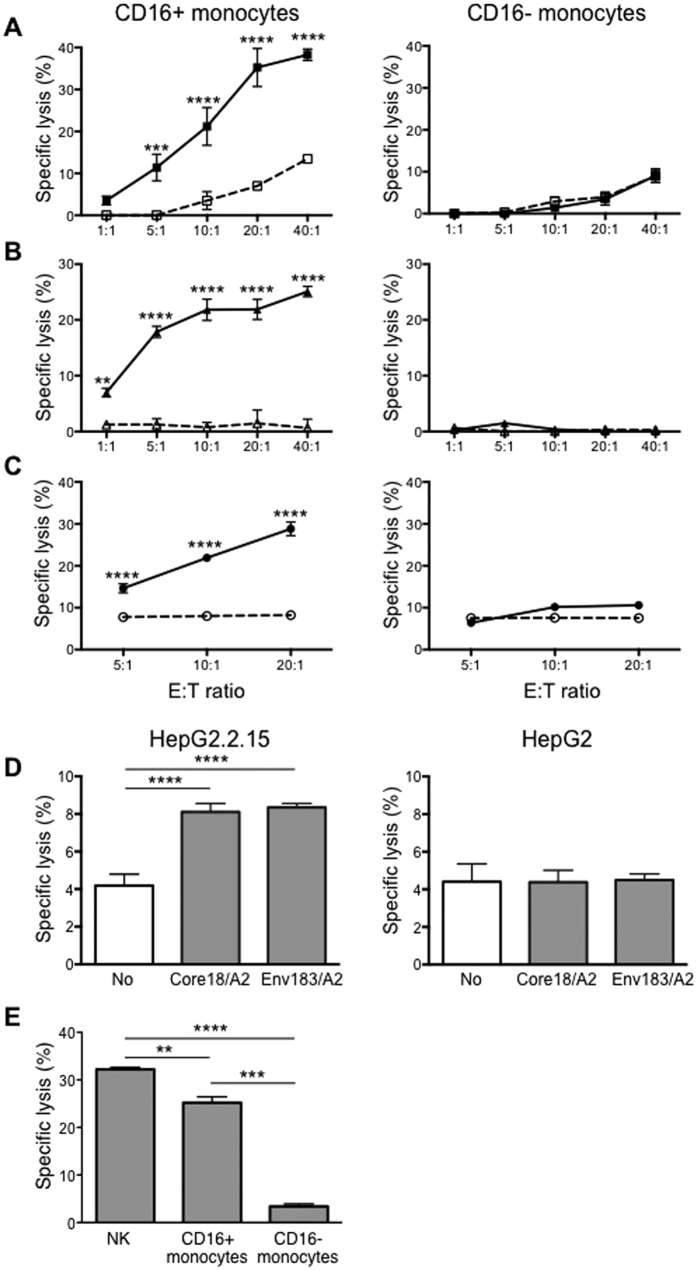
CD16+ but not the CD16− monocytes are able to perform ADCC on therapeutic antibody–coated tumour and virus-infected cell lines. ADCC by CD16+ (left panel) and CD16− (right panel) monocytes on A549 lung adenocarcinoma **(A)** Raji Burkitt’s lymphoma **(B)** and SKBR3 breast adenocarcinoma **(C)** at the indicated effector to target (E:T) cell ratios. Tumour cell lines were either uncoated (open symbols and dotted lines) or pre-coated with the respective therapeutic antibodies αGM2 (KM966), αCD20 (Rituximab) and αHER2 (Trastuzumab) (closed symbols and solid lines). Data shown are representative data of at least 5 independent experiments and plotted as mean ± SD of triplicate wells for each respective experiment. **p ≤ 0.01, ****p ≤ 0.0001 with respect to uncoated target cells at the respective E:T ratios based on Two-way ANOVA (****p ≤ 0.0001). **(D)** ADCC by CD16+ monocytes on Hepatitis B virus-infected cell line HepG2.2.15 (left) or parental HepG2 (right) cells at E:T ratio of 10:1. Both HepG2 cell lines were either uncoated (white bar) or coated with TCR-like antibodies recognising core or envelope peptides respective (grey bars). Data shown are plotted as mean ± SD; n = 4. ****p ≤ 0.0001 with respect to uncoated cells based on One-way ANOVA (****p ≤ 0.0001). (**E**) NK cells, CD16+ and CD16− monocytes isolated from the same individual were co-cultured with KM966-coated A549 at an E:T ratio of 10:1. Data plotted is mean ± SD, n = 2. **p ≤ 0.01, ***p ≤ 0.001 and ****p ≤ 0.0001. One-way ANOVA (***p ≤ 0.001).

When A549, Raji and SKBR3 target cells were co-cultured with either CD16− or CD16+ monocytes for 4 hours in ADCC assay, minimal target cell lysis was detected (Fig. 1A–C; dotted lines). However, when target cells were pre-incubated with 10 μg/ml of their respective antibodies, the CD16+ monocytes lysed between 10% and 40% of the cells, depending on the E:T ratio and cancer cell line used (Fig. 1A–C; left panels solid lines). Percentage specific lysis also increased with increasing E:T ratios. In contrast, co-culture of CD16− monocytes with antibody-coated target cells did not result in increased lysis compared to uncoated target cells (Fig. 1A–C; right panels solid lines), indicating that CD16− monocytes lack ADCC capacity.

To determine if CD16+ monocytes could also lyse antibody-coated viral-infected cells, we set up an ADCC assay with the HepG2.2.15 cell line. HepG2.2.1.5 is a stable HBV-DNA transfected hepatoma cell line producing HBV virions and HBV antigens. They are able to present potentially all HLA-A201 restricted HBV peptides and are frequently used in HBV infection studies^25^. Two TCR-like antibodies, Env183/A2 mAb and Core18/A2 mAb that specifically recognize the HBV envelope epitope (Env at positions 183 to 191; Env183-191) or core epitope (core at positions 18 to 27; Core18–27) presented on the HLA-A201 molecule respectively were used^26^. In the absence of TCR-like antibody coating, the specific lysis of HepG2.2.15 by CD16+ monocytes was <4%. The specific lysis increased by 2-fold to >8% when HepG2.2.15 cells were pre-coated with either one of the two TCR-like antibodies (Fig. 1D, left panel). No such enhancement in specific lysis was observed when the parental HepG2 cells with TCR-like antibodies (Fig. 1D, right panel) was used indicating that only CD16+ monocytes target HBV-infected cells coated with specific antibodies.

The specificity of ADCC was confirmed by experiments where target cells were pre-incubated with an antibody not reactive against the cells. Thus, lysis of CD20− A549 cells was similar, regardless of whether rituximab was present (Supplementary Figure 1B, open square symbol). To compare the potency of cytotoxicity of CD16+ monocytes to NK cells, we isolated NK cells, CD16+ and CD16− monocytes from the same individual and measured lysis of KM966-coated A549 cells at an E:T ratio of 10:1. CD16+ monocytes lysed 25% of KM966-coated A549 cells and the specific lysis by NK cells were 32%. As before, CD16− monocytes exhibited minimal ADCC activity at 3% specific lysis (Fig. 1E).

### CD16+ monocytes can exert ADCC on primary leukemic cells

**Figure 2:**
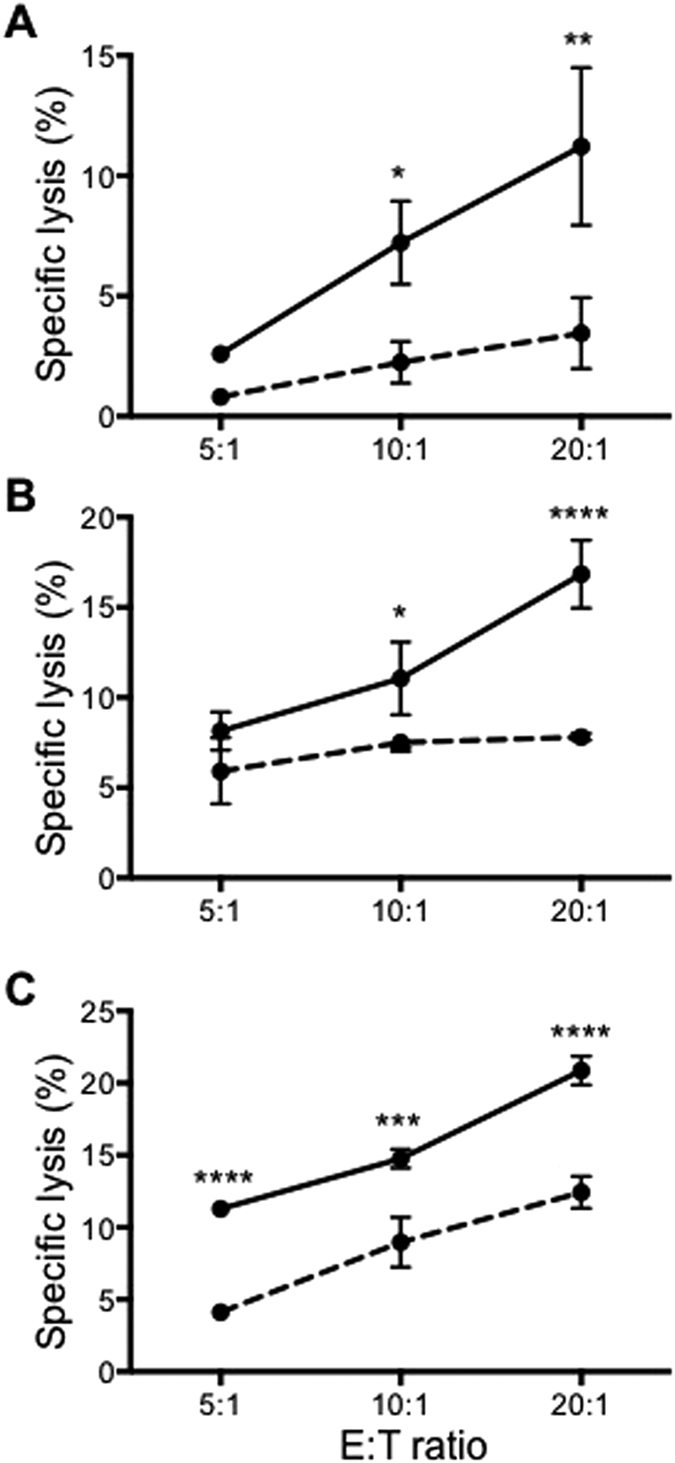
CD16+ monocytes from healthy individuals are able to perform ADCC on Rtx-coated primary B leukemic cells. ADCC by CD16+ monocytes from three healthy individuals on primary B leukaemic cells isolated from three B-CLL patients (**A–C**) either uncoated (dotted lines) or coated with Rtx (solid lines) at the indicated E:T ratios. All data plotted are mean ± SD of triplicate wells for each experiment. *p ≤ 0.05, **p ≤ 0.01, ***p ≤ 0.001 and ****p ≤ 0.0001 with respect to uncoated leukaemic cells at the respective E:T ratios and based on Two-way ANOVA (****p ≤ 0.0001).

Rituximab is commonly used for the treatment of B-cell non-Hodgkin lymphoma and chronic lymphocytic leukaemia (B-CLL)^27^,^28^. We assessed the capacity of CD16+ monocytes to exert ADCC against primary CD20+ leukemic cells from patients with B-CLL. Leukemic cells were isolated from either bone marrow (Fig. 2A,B) or peripheral blood (Fig. 2C) from three B-CLL patients. These cells were co-cultured at various E:T ratios with CD16+ monocytes isolated from healthy donors and substantial lysis of the CD20+ primary B-CLL cells in an antibody-dependent manner was observed (Fig. 2; solid lines versus dotted lines).

### CD16 is indispensible for ADCC by monocytes

**Figure 3:**
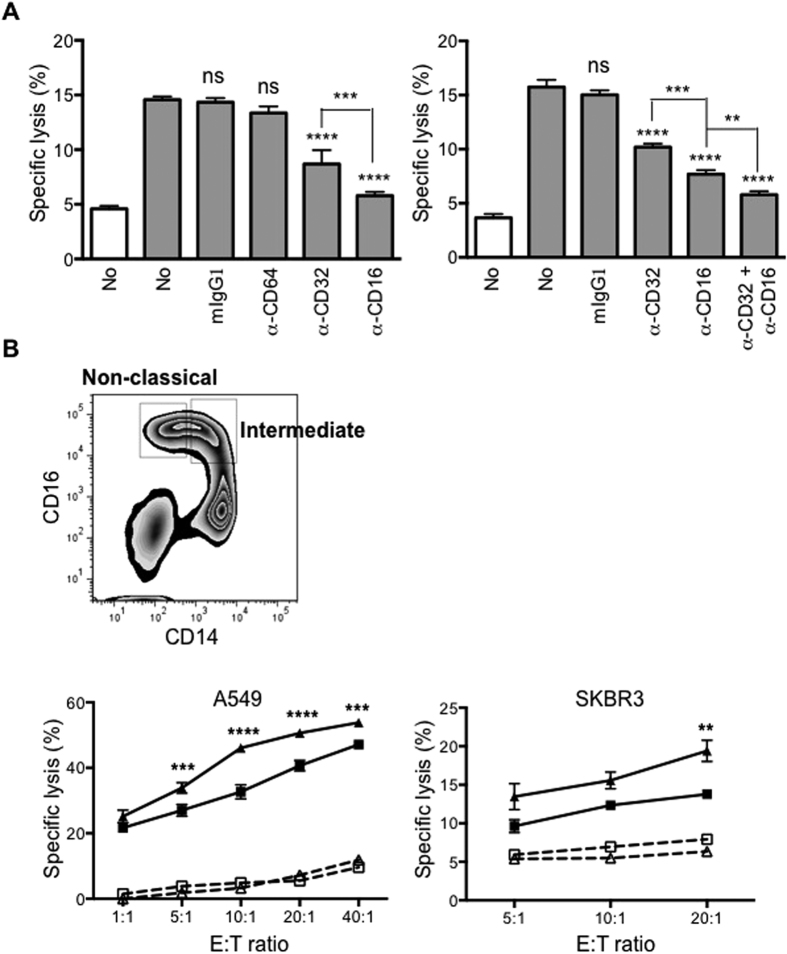
FcγRIII is involved in CD16+ monocyte mediated ADCC. **(A)** CD16+ monocytes were untreated or pre-treated with FcγR blocking antibodies or isotype control and co-cultured with uncoated (white bar) or trast-coated SKBR3 (grey bar), n = 3. **p ≤ 0.01, ***p ≤ 0.001. ****p ≤ 0.0001 is compared to untreated trast-coated SKBR3. One-way ANOVA (****p ≤ 0.0001), ns = not significant. **(B)** CD16+ monocytes were FACS-sorted to intermediate and non-classical subsets (FACS plot). Non-classical monocytes (triangle symbol) exhibit higher ADCC than intermediate monocytes (square symbol) on A549 (left graph) and SKBR3 (right graph) pre-coated with respective antibody (closed symbols) at various E:T ratios. Uncoated target cells are represented by open symbols. Data shown are representative of 2 independent experiments for each tumour cell lines. **p ≤ 0.01, ***p ≤ 0.001 and ****p ≤ 0.0001 compared to intermediate monocytes at the respective E:T ratios. Two-way ANOVA (****p ≤ 0.0001). **(C)** Histogram plot showing SLAN expression on non-classical monocytes (left). SLAN+ and SLAN− monocytes were co-cultured with uncoated (white bar) or trast-coated SKBR3 (grey bar), n = 3. One-way ANOVA (***p ≤ 0.001) (right). **(D)** CD16− monocytes were untreated or pre-treated with M-CSF, TGF-β or IL-10. The rMFI of CD16 was determined by subtracting mean fluorescence intensity (MFI) of isotype-matched control (dashed line) from the CD16 labelling (solid line). Percentages indicate positively-stained cells. Bar graph depicts ADCC assay performed using untreated and treated CD16− monocytes with trast-coated SKBR3. Data plotted as fold difference with respect to specific target lysis of freshly isolated CD16− monocytes, which was 5%, n = 7. *p ≤ 0.05, ***p ≤ 0.001 compared to untreated CD16− monocytes and One-way ANOVA (****p ≤ 0.0001). **(E)** CD16− monocytes were either mock or CD16 mRNA transfected. Histogram plots showing CD16 expression either labelled with CD16 antibody (solid line) versus isotype-matched control (dashed line). Percentages indicate positively stained cells. ADCC assay was performed using mock and CD16 mRNA-transfected CD16− monocytes co-cultured with trast-coated SKBR3 cells (bar graph), n = 3. **p ≤ 0.01 based on Student’s *t* test. All the ADCC assays are based on E:T ratio of 10:1 and all data are plotted as mean ± SD.

As CD16+ monocytes also express high levels of CD32 and low levels of CD64 (two other Fcγ receptors), we determined their relative role in ADCC exerted by CD16+ monocytes. Minimal inhibition of ADCC activity was observed when isotype control mouse IgG1 antibody or CD64 blocking antibody was added (Fig. 3A; left panel). A 2-fold inhibition in lysis was observed when 10 μg/ml of CD32 blocking antibody was added. However, the addition of 10 μg/ml of CD16 blocking antibody inhibited target cell lysis to levels similar to basal cytotoxicity (Fig. 3A; left panel). Even when a lower concentration of CD32 or CD16 blocking antibodies (5 μg/ml) was used, the extent of inhibition was consistently higher when CD16 was blocked (Fig. 3A; right panel). However, ADCC was further inhibited when 5 μg/ml of CD32 and CD16 blocking antibodies were added together (Fig. 3A; right panel).

CD16+ monocytes can be further subdivided according to their expression of CD14: CD14high (intermediate subset) or CD14low (non-classical subset), as depicted in FACS plot in Fig. 3B. When the ADCC assay was performed using either A549 or SKBR3 cells as targets, both the intermediate and non-classical subsets exhibited ADCC activities on antibody-coated target cells (Fig. 3B; solid lines, left and right panels respectively). The non-classical subset can also be further subdivided based on SLAN expression into SLAN− and SLAN+ monocytes (Fig. 3C; left panel). A previous study reported that human blood dendritic cells stained positive for SLAN exhibited potent ADCC activity against antibody coated targets^29^. Hence, we determined whether SLAN+ monocytes would exhibit a higher ADCC activity than SLAN− monocytes in our system. SLAN+ and SLAN− monocytes lysed 15% ± 3% and 13% ± 2% of trastuzumab-coated SKBR3 cells respectively at an E:T ratio of 10:1 (Fig. 3C; right panel), indicating that ADCC was not exclusively from the SLAN+ monocytes. This indicated that different monocyte subsets that express CD16 exhibited similar ADCC activities.

CD16− monocytes did not exert ADCC. We assessed whether enforced CD16 expression would induce ADCC capacity. Treatment of CD16− monocytes with M-CSF, TGF-β or IL-10, previously reported to induce surface expression of CD16^30^,^31^,^32^ induced surface expression of CD16 to varying extents, with the relative mean fluorescence intensity (rMFI) and percentage CD16 positive cells being the highest when treated with IL-10 (rMFI: 6557; 53.8%), followed by TGF-β (rMFI: 4288; 46.1%) and M-CSF (rMFI: 3176; 32.4%) (Fig. 3D; histograms). Untreated CD16− monocytes cultured for the same period also slightly up-regulated CD16 expression due to their endogenous production of a low level of M-CSF (rMFI: 2216; 17.2%). Using freshly-isolated CD16− monocytes from the same donor as a control, we tested ADCC with SKBR3 cells as target and observed an increase in specific ADCC over baseline for both treated and untreated monocytes (Fig. 3D; bar graph). The percentages of specific lysis positively correlated with the induced CD16 surface expression level on the monocytes.

To further confirm the relationship between CD16 expression and ADCC activity, we ectopically expressed CD16 on the CD16− monocytes by mRNA electroporation. To minimize the level of spontaneously up-regulated CD16 expression on the cultured CD16− monocytes, we tested and selected the 10 hrs post-transfection time point as ≥60% of the transfected monocytes were positive for CD16 expression while ≤10% of the mock transfected monocytes showed CD16 expression (Fig. 3E; histograms). CD16 mRNA-transfected monocytes when co-cultured with trastuzumab-coated SKBR3 cells at an E:T ratio of 10:1 exhibited a significant increase in specific lysis of 12% ± 4% as compared to mock-transfected cells (8% ± 2%) (Fig. 3E; bar graph).

### ADCC by CD16+ monocytes requires cell-cell contact and involved β2-integrins

**Figure 4:**
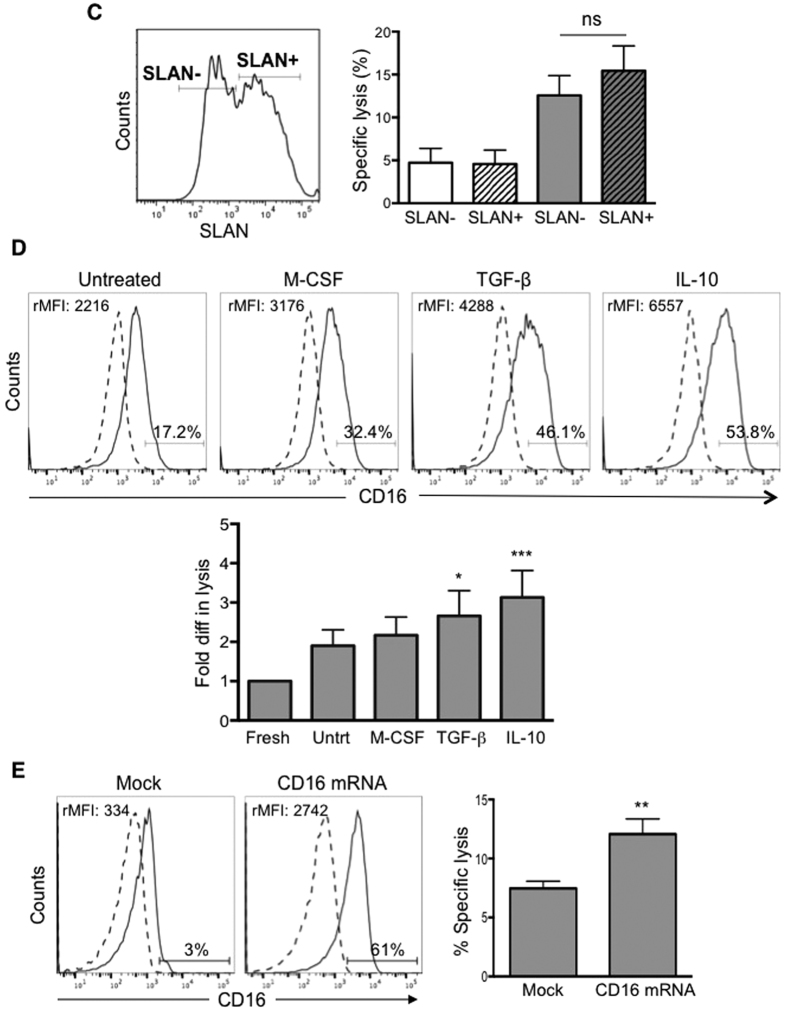
Cell-cell contact mediated through β2-integrins is needed for ADCC to take place. **(A)** CD16+ monocytes were co-cultured with SKBR3 at E:T ratio of 10:1. The x-axis depicts the conditions at which tumour targets were used. SKBR3 cells labelled with BATDA are depicted with black diamond inside and are either uncoated or trast-coated (antibody symbol). Data plotted are mean ± SD, n = 3. ****p ≤ 0.0001 with respect to trast-coated SKBR3 and based on One-way ANOVA (****p ≤ 0.0001), ns = not significant. **(B)** ADCC by CD16+ monocytes was assessed using time-lapsed imaging (see also Supplementary Movie). Interaction of CD16+ monocyte (black arrow) and trast-coated SKBR3 (dotted) was tracked at 20 secs intervals for up to 2 hrs and static images at various times as indicated are shown. Brightfield images were visualised under a FV-1000 confocal system with an inverted Olympus IX81 microscope at 200x magnification using Fluoview software FV10-ASW 2.0. Data shown is a representative of 3 independent experiments where >10 different cells were followed in each experiment**. (C)** CD16+ monocytes were either untreated or pre-treated with blocking antibodies for the indicated integrins prior to co-culturing with either uncoated (white bar) or trast-coated SKBR3 (grey bar) at E:T ratio of 10:1. Data plotted are mean ± SD, n = 3. ****p ≤ 0.0001 with respect to untreated trast-coated SKBR3 based on One-way ANOVA (****p ≤ 0.0001), ns = not significant.

To determine whether ADCC by CD16+ monocytes required direct contact with target cells, we tested ADCC against mixtures of trastuzumab-coated and uncoated SKBR3 cells labeled with BATDA. We detected release of the BATDA label when the antibody-coated cells were the ones pre-labeled, and not when the non-coated cells were carrying the BATDA (Fig. 4A). Thus, cells lacking antibody coating were not lysed by CD16+ monocytes, even when in the same culture as antibody-coated cells that were being actively lysed.

The requirement for effector-target cell contact was further confirmed by time-lapse microscopy. CD16+ monocytes were added to adherent SKBR3 cells pre-incubated with trastuzumab and the interactions were visualized over 1.5 hours (Supplementary Movie 1). Selected images from the movie are depicted in Fig. 4B and showed that a CD16+ monocyte (black arrow) approached and contacted an adherent SKBR3 cell (outline with dotted line). The interaction became more intense with time and the SKBR3 cell began to detach from the substratum at 30 mins. By 1 hour, lytic cell death of the target became evident (Fig. 4B).

Since cell-cell contact is required for ADCC to occur, we asked if integrins might be involved by using blocking antibodies against different integrin molecules. Minimal inhibition of ADCC activity was observed when CD29 blocking antibody was added (Fig. 4C). A significant reduction in lysis of target cells was observed when blocking antibodies against CD11a, CD11b, CD11c and CD18 were added (Fig. 4C). This indicated that β2- and not β1-integrin is involved in the ADCC activity by CD16+ monocytes.

### ADCC by CD16+ monocytes is mediated through TNFα

**Figure 5:**
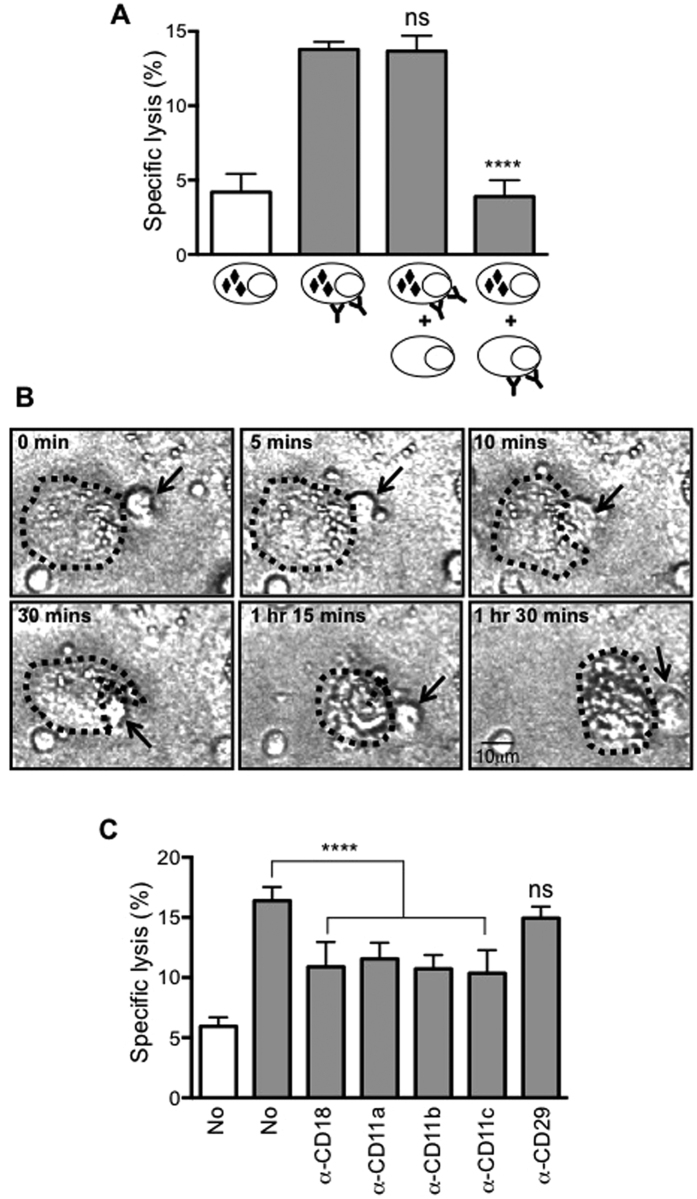
CD16+ monocytes perform ADCC through TNFα. **(A)** CD16+ monocytes were co-cultured with either uncoated (white bar) or KM966-coated A549 (grey bar) at E:T ratio of 10:1 in the absence or presence of blocking antibodies. Data are plotted as mean ± SD, n = 4. ****p ≤ 0.0001 with respect to KM966-coated A549 based on One-way ANOVA (****p ≤ 0.0001), ns = not significant. **(B)** SKBR3 cells were FACS sorted into TNFRlo and TNFRhi populations, coated with Trast and co-cultured with CD16+ monocytes. Data are plotted as mean ± SD, n = 3. **p ≤ 0.01 with respect to parental SKBR3 and based on One-way ANOVA (****p ≤ 0.0001). **(C)** Raji cells were either uncoated (white bar) or pre-coated with Rtx (grey bar) before fixing with 1% PFA and co-cultured with CD16+ monocytes for 4 hours. Supernatant was collected for TNFα ELISA. Data are plotted as mean ± SD, n = 3. ****p ≤ 0.0001 based on Student’s *t* test. **(D)** SKBR3 (top panel) and primary B-CLL cells (bottom panel) were pre-coated with Trast and Rtx respectively and treated with the indicated concentrations of recombinant human TNFα (rhTNFα) in the absence (No Fc) or presence (+ Fc) of anti-human IgG. Data shown are representative of 2 independent experiments for SKBR3 and 3 independent experiments for primary B-CLL cells and plotted as specific lysis with respect to untreated cells. **p ≤ 0.01 and ****p ≤ 0.0001 with respect to no Fc of the respective E:T ratios and based on Two-way ANOVA (****p ≤ 0.0001). **(E)** SKBR3 (top histograms) and primary B-CLL cells (bottom histograms) were pre-coated with Trast and Rtx respectively and treated with 5 µg/ml rhTNFα in the absence (No Fc) or presence (+ Fc) of anti-human IgG. Histogram plots of TNFR expression labelled with TNFR antibody (black solid line) versus isotype-matched control antibody (grey dashed line). The rMFI of TNFR was determined by subtracting MFI of isotype-matched control from the TNFR antibody labelling. Percentages indicate the proportion of positively stained cells. Data shown are representative of 1 experiment for SKBR3 and 3 independent experiments for primary B-CLL cells.

Monocytes/macrophages can promote cytotoxicity through several mechanisms, such as the release of reactive oxygen species (ROS), reactive nitrogen species (RNS) and TNFα^33^,^34^. To clarify the mechanism utilized by CD16+ monocytes to lyse antibody-coated targets, we added inhibitors and blocking antibodies to the ADCC assay. Blocking ROS with N-acetyl-cysteine (NAC) and NOS with NG-monomethyl-L-arginine (LMMA) did not inhibit the ability of CD16+ monocytes to exert ADCC (data not shown). However, the addition of blocking antibodies against both TNFα and TNF receptor (TNFR) significantly inhibited the lysis of antibody-coated A549 lung adenocarcinoma cells (Fig. 5A). Minimal inhibition of ADCC activity was observed when isotype control mouse IgG1 antibody was added. We further established that TNFR expression on the target cells is required in the ADCC by CD16+ monocytes through assessing the lysis of TNFR high-expressing (TNFRhi) and low-expressing (TNFRlo) SKBR3 cells sorted from the parental cell line. In comparison to the parental SKBR3 cells, a significantly greater proportion of the TNFRhi cells were lysed and a significant reduction in lysis was observed for the TNFRlo cells (Fig. 5B). Furthermore, CD16+ monocytes secreted significantly higher amount of TNFα when co-cultured with antibody-coated versus uncoated Raji cells (Fig. 5C). These data indicated that interaction of CD16+ monocytes with antibody-coated target cells induced their production of TNFα, which in turn binds to TNFR-expressing target cells to promote their cell death via a TNFα-mediated mechanism.

As shown in Fig. 4A, only antibody-coated target cells in direct contact with CD16+ monocytes were lysed while neighboring cells were not affected by TNFα secreted by the monocytes. We were interested to determine if the clustering of antibodies bound to the target cells were rendering them susceptible to TNFα-mediated cell death. To address this, we added Fcγ-specific IgG antibody to induce cross-linking of the antibody bound on the target cells so as to mimic antibody clustering upon interaction with CD16 molecules on the monocytes. In the condition where Fcγ-specific IgG antibody was added to induce cross-linking of trastuzumab-coated SKBR3 cells, addition of exogenous rhTNFα induced target cell lysis in a dose-dependent manner (Fig. 5D; top panel grey striped bars). No enhancement in lysis was observed with increasing amount of TNFα in the absence of Fcγ-specific IgG antibody or when the SKBR3 cells were not coated with trastuzumab (Fig. 5D; top panel grey bars and Supplementary Figure 2A; top panel respectively). A similar observation was made with primary B-CLL cells and rituximab (Fig. 5D, bottom panel and Supplementary Figure 2A, bottom panel). Clustering of specific antibody bound to these target cells promoted their susceptibility to TNFα because the expression of TNFR was greatly increased on these cells upon cross-linking (Fig. 5E; right histograms). A slight up-regulation of TNFR expression could be observed in the absence of cross-linking while no up-regulated expression of TNFR was observed on uncoated target cells (Fig. 5E; left histograms and Supplementary Figure 2B respectively).

### Pre-stimulation can enhance the ADCC by CD16+ monocytes

We screened a panel of stimuli to assess whether pre-treatment of CD16+ monocytes might enhance their ADCC ability. Alongside IL-12 and IL-15, factors known to enhance NK cell cytotoxicity^35^, we also tested several other factors known to activate monocytes/macrophages: the pro-inflammatory cytokine IFNγ, TLR agonists LPS and R848, and the endogenous danger signals HMGB1 and S100A9.

**Figure 6:**
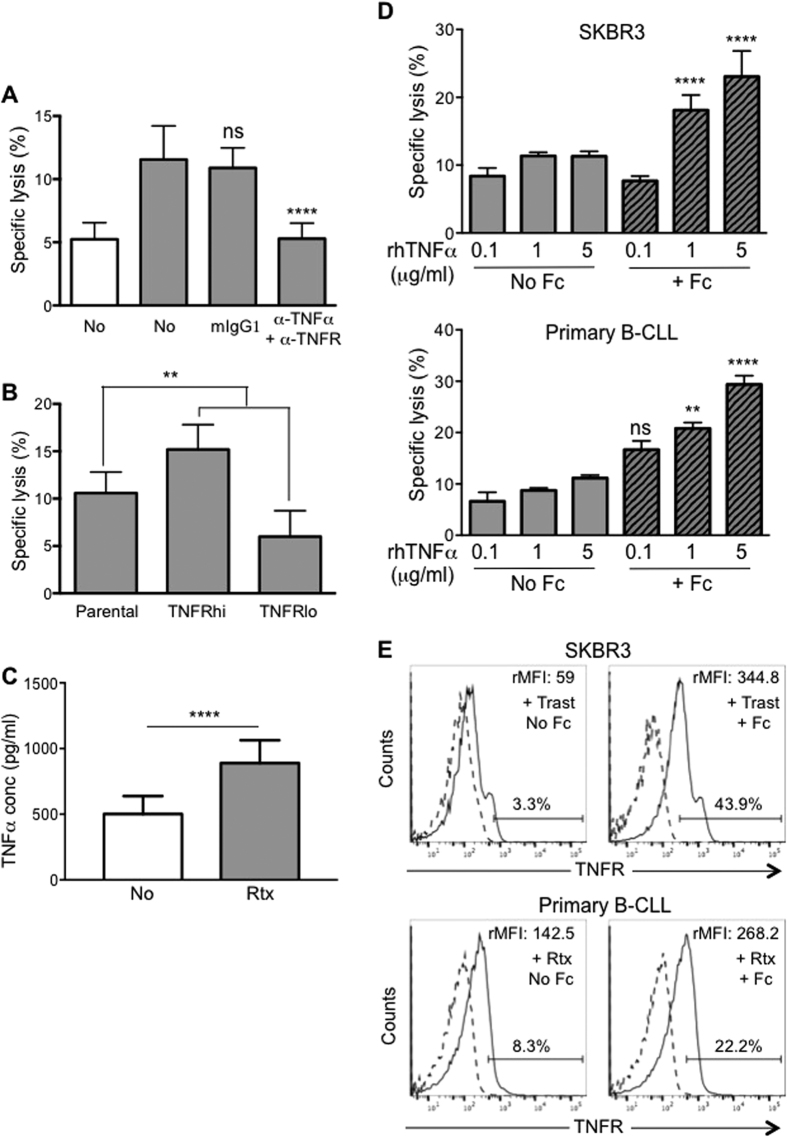
Pre-treatment with various stimulus enhances the ability of CD16+ monocytes to perform ADCC. **(A–C)** CD16+ monocytes, NK and CD16− monocytes respectively were either untreated (white bar) or pre-treated with various stimulus (grey bar). And then co-cultured with KM966-coated A549 at E:T ratio of 10:1. Data was plotted as fold difference in target cell lysis of treated to untreated effector cells. The percentage specific lysis of untreated CD16+, NK and CD16− cells were 10.1% (±4.6%), 20.6% (±10.5%) and 3.7% (±0.7%) respectively. Data are plotted as mean ± SD, n = 3. *p ≤ 0.05 and ****p ≤ 0.0001 with respect to untreated effector cells and based on One-way ANOVA (****p ≤ 0.0001 for CD16+ and NK), ns = not significant.

NK cells, CD16+ and CD16− monocytes from the same donor were pre-treated in parallel with the various stimuli before co-culture with KM966-coated A549 cells to assess ADCC. NK cells pre-treated with IL-12 and IL-15 exhibited an almost 2.5-fold increase in lysis of KM966-coated A549 over untreated NK cells. However, no enhancement in ADCC activity was observed for either CD16+ or CD16− monocytes pre-treated with IL-12 and IL-15 (Fig. 6). In contrast, CD16+ monocytes pre-treated with LPS, R848, IFNγ, S100A9 or HMGB1 showed significantly increased ADCC potency compared to untreated CD16+ monocytes (Fig. 6A). IFN-γ was the most potent inducer of ADCC activity by CD16+ monocytes. The expression of CD64, CD32a(FcγRIIa) and CD32b(FcγRIIb) on CD16+ monocytes treated with the various stimuli were not significantly altered apart from an increased in CD64 expression upon treatment with IFNγ (Supplementary Figure 3). Interestingly, all the stimuli that could enhance ADCC by CD16+ monocytes did not have any effect on ADCC exerted by NK cells or CD16− monocytes (Fig. 6B,C), nor did they enhance the capacity of CD16+ monocytes to promote antibody-independent cytotoxicity (data not shown).

### CD16+ monocytes from B-CLL patients have ADCC function

**Figure 7:**
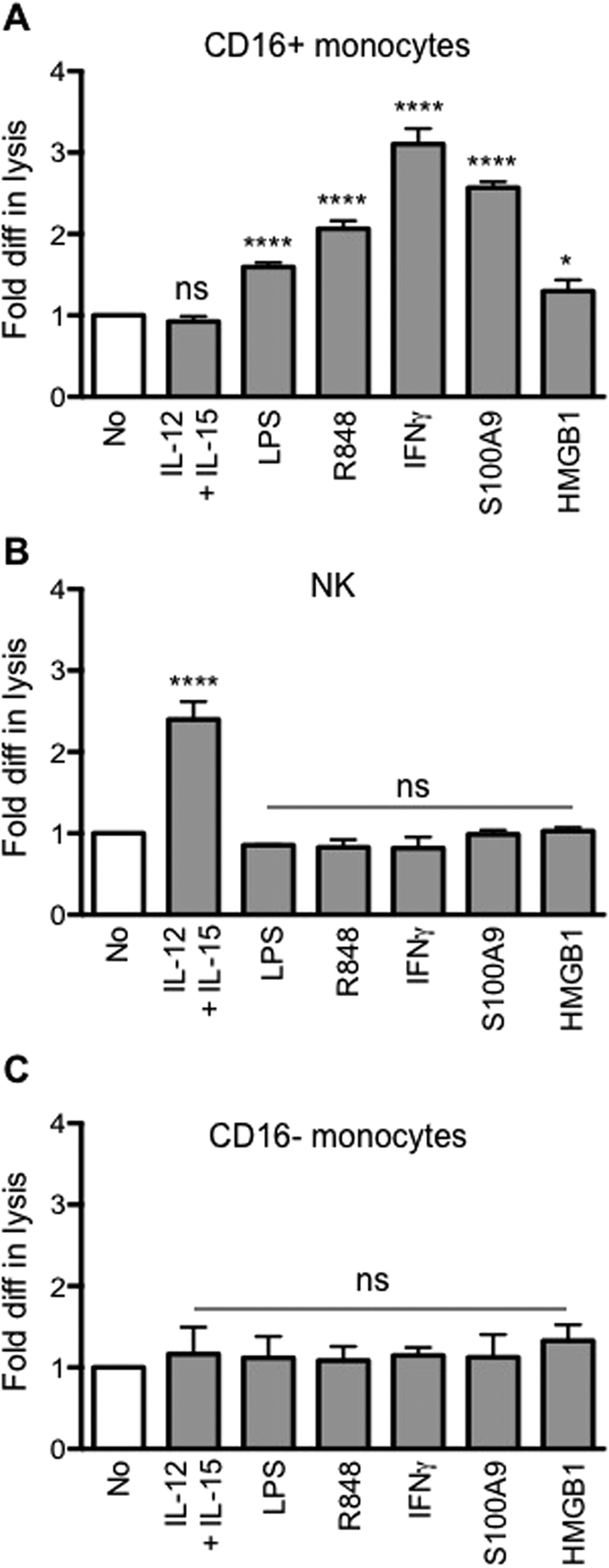
CD16+ monocytes from B-CLL patients exhibit ADCC activity similar to healthy individuals. **(A)** CD16+ monocytes (left graph) or NK cells (right graph) isolated from the same leukemia patient (striped) or healthy donor (solid) were co-cultured with either uncoated (white bar) or Trast-coated SKBR3 (grey bar) at E:T ratio of 10:1. Data are plotted as mean ± SD, n = 2. ***p ≤ 0.001 based on One-way ANOVA (****p ≤ 0.0001). **(B)** CD16+ monocytes (left graph) or NK cells (right graph) isolated from the same patient (striped) or healthy donor (solid) were co-cultured with either uncoated (white bar) or Rtx-coated primary B-CLL cells (grey bar) at E:T ratio of 10:1. Data are plotted as mean ± SD, n = 4. **p ≤ 0.01 based on One-way ANOVA (****p ≤ 0.0001).

NK cells from cancer patients are known to be dysfunctional, i.e. exhibit reduction in ADCC ability^15^,^16^,^17^. We isolated CD16+ monocytes and NK cells from healthy individuals and B-CLL patients and performed ADCC assays using either SKBR3 cell lines or primary B-CLL cells as target cells. The primary B-CLL cells used as target were isolated from patients that were different from those whom NK and CD16+ monocytes were purified. We observed that CD16+ monocytes from patients were able to lyse a significantly higher proportion of trastuzumab-coated SKBR3 cell lines and rituximab-coated primary B-CLL cells as compared to target cells not bound with antibodies (Fig. 7A,B; left panels striped bar respectively). Similar to previous reports, NK cells from B-CLL patients were also able to perform ADCC on both trastuzumab-coated SKBR3 cell lines and rituximab-coated primary B-CLL cells. However, in comparison to healthy individuals, NK cells isolated from B-CLL patients lysed a significantly lower percentage of both trastuzumab-coated SKBR3 cells and rituximab-coated primary B-CLL cells as compared to NK cells from healthy individuals (Fig. 7A,B; right panels respectively). Unlike NK cells, CD16+ monocytes isolated from the same patients could lyse antibody-coated target cells as efficiently as CD16+ monocytes from healthy individuals for both SKBR3 cells and primary B-CLL cells (Fig. 7A,B; left panels respectively).

## Discussion

Our data show that the human blood monocyte subsets that express CD16 possess the capacity to exert ADCC on cell lines, primary tumor cells and virally infected cells. ADCC by CD16+ monocytes was as efficient as that of NK cells. The CD16− subset when acquired CD16 expression could promote ADCC, revealing that this subset intrinsically possessed the machinery required to promote cytolysis of antibody-coated targets. ADCC activity could be further enhanced upon stimulation of CD16+ monocytes with TLR agonists, cytokines such as IFNγ and DAMPs. Cell-cell contact was essential and target cells lysis occurred through a TNFα-mediated mechanism. CD16+ monocytes from B-CLL patients did not exhibit discernible dysfunctions and showed ADCC activity similar to that of CD16+ monocytes from healthy individuals.

The involvement of other immune cell types in mediating ADCC has been clearly evident in numerous preclinical studies. These mouse studies demonstrated that monocytes and/or macrophages and not NK cells are the principal mediators of ADCC against α-CD20-coated B cells *in vivo*^4^,^19^ further supporting the importance of monocyte in eradicating antibody-coated cells *in vivo*.

Our observation that the capacity for ADCC is unique to the CD16+ subset of human monocytes, is in line with recent findings both in humans^36^ and in mice^19^. Mice deficient in FcγRIV, the murine homolog of human CD16^37^ exhibit defects in several models of ADCC^38^. In our study, the engagement of CD16 and potentially CD32, but not CD64, was necessary to trigger ADCC, which was similar to that reported for SLAN+ DC^29^. However, unlike the data by Schmitz *et al*.^29^ showing equal contribution of both CD32 and CD16 to ADCC by DC, our study showed that blocking CD16 inhibited lysis to a greater extent than CD32. CD16− monocytes were unable to exert ADCC despite the expression of CD64 and CD32 unless CD16 expression was enforced. Their differential ADCC could not be explained by the expression of CD32 isoforms, i.e. CD32a (activating) and CD32b (inhibiting) since CD32a expression is similar on both monocyte subsets and CD32b expression is higher on CD16+ monocytes^20^,^39^ and unpublished data. Furthermore, the fact that the level of ADCC activity of these cells positively correlated with the level of CD16 expression further confirms the essential role played by CD16. Functional polymorphisms in the coding regions of the different FcγR s are known to impact their affinity for IgG. In fact, many studies correlating FcγR polymorphisms, particularly for CD32 and CD16 with clinical response, suggest a role for FcγR-mediated effector functions in antibody therapy. In both rituximab and trastuzumab treatments for follicular lymphoma and metastatic breast cancer respectively, polymorphism in both CD16 (i.e. FcγRIIIa-158V/F) and CD32 (i.e. FcγRIIa -131H/R) were shown to correlate with clinical responses^1^,^11^. Another study in metastatic breast cancer found homozygosity for FcγRIIa-131H alone to be significantly associated with a stronger anti-tumour response and progression free survival when patients are treated with trastuzumab^40^. These further support a predominant role of myeloid cells including monocytes in antibody therapy.

Panitumumab, an EGF receptor antibody, currently the only approved human IgG2 antibody, has been shown to promote ADCC by myeloid cells including monocytes as effectively as the IgG1 antibody at low doses^41^. Unlike IgG1, they bind CD32 with higher affinity^42^. With our study demonstrating that CD32 together with CD16 are involved in ADCC provide support for the potential application of IgG2 antibody in immunotherapy.

Impaired NK cell function has been reported in various types of malignancy. Alterations in the expression of activating and inhibiting receptors, increased MHC class I expression, down-regulated expression in the signal transducing ζ chain, CD16 and cytotoxic machinery were reported to contribute to NK cell dysfunction^15^,^16^,^17^. Although reduced expression of CD16 on NK cells was commonly observed in many malignancies, there was no significant down-regulation of CD16 expression on NK cells from B-CLL patients in our study (data not shown). Nevertheless, these cells still exhibited a reduced ADCC ability compared to NK cells from healthy individuals possibly due to other factors mentioned above. On the contrary, CD16+ monocytes from these patients were as capable as CD16+ monocytes from healthy individuals in terms of ADCC.

Unlike NK cells where IL-12 and IL-15 activates and enhances their cytolytic ability, the ADCC capacity of CD16+ monocytes was unaffected by these cytokines. A previous study showed that IFNγ could enhance monocyte/macrophage ADCC activity but only via FcγRI^43^. While TLR agonists such as CpG can enhance the cytolytic ability of NK cells^44^, LPS and R848, which ligate TLR4 and TLR7/8 respectively, specifically enhanced the ADCC activity of CD16+ monocytes. R848 has been shown to activate NK cell cytotoxicity after 18 hrs^45^ but no enhancing effect was detected at the 5 hr time point used in our study. TLR8 agonist, in particular, promoted ADCC by monocytes through a IL-12-induced granzyme B expression and secretion after 12 hrs^46^. However, no granzyme B protein was detectable when CD16+ monocytes were treated with R848 for 5 hrs in our study (data not shown) and IL-12 treatment also did not enhance ADCC at this time-point. Both HMGB1 and S100A9 are self-derived molecules well-known as damage-associated molecular patterns (DAMPs), which are released at sites of tissue damage or regions of necrotic cells^47^. Treatment of cancer with therapeutic antibodies is routinely performed in conjunction with chemotherapy or surgery, which leads to tissue damage and death in the tumour environment. As such, monocytes recruited to the tumour site and activated locally by DAMPs might then be able to promote killing of the remaining cancer cells coated with the therapeutic antibody. Both HMGB1 and S100A9 as well as TLR agonists and IFNγ could also activate monocytes to release pro-inflammatory cytokines including TNFα^48^,^49^,^50^. Specifically, IL-10 and TGF-β over-production were shown to decrease NK cell mediated functions including ADCC, down regulation of CD16 expression and IFNγ production^16^,^51^,^52^, our data however showed that the enhancement of CD16 expression on CD16− monocytes by these mediators conferred these monocytes with ADCC activity, which might be favorable for cancer immunotherapy.

Previous studies showed that FcγR engagement can induce β2-integrin activation on murine macrophages for optimal phagocytic activity but played no role in ADCC by *in vitro* differentiated human macrophages^53^,^54^. However, β2-integrin appears to be involved in ADCC by CD16+ monocytes in our study. Besides promoting the release of proinflammatory cytokines, stimuli like LPS and S100A9 may potentially be enhancing ADCC activity of CD16+ monocytes through regulating the activity of CD11b, the binding partner of β2-integrin^55^,^56^.

The production of TNFα by activated macrophages and monocytes has been well described. The involvement of TNFα in ADCC by macrophages through antibody neutralization assay had also been reported in numerous studies^29^. Nevertheless, the exact mechanism is still unclear. The TNFα secreted by CD16+ monocytes upon engagement of the FcγR could be involved in the activation of b2-integrins in an autocrine fashion similar to that reported for neutrophils^57^. In addition, as shown for breast cancer cells, the secreted TNFα also induced ICAM1 expression on the tumor cells in our study (data not shown)^58^. Together, these would result in further cell-cell interaction to promote target cell lysis. Most importantly, only target cells in direct contact with the CD16+ monocytes will undergo ADCC because the clustering of antigens on the target cell surface through engaging the FcγR on the CD16+ monocytes promoted TNFR surface expression, predisposing these target cells to TNFα-mediated cell death. A finding that has not yet been reported.

Moreover, CD16+ monocytes have been reported to expand during infection, autoimmune disease and certain cancers such as colorectal, gastric and breast^59^,^60^. It will therefore be interesting to understand how this biological observation might link with clinical outcomes, and in particular whether higher numbers of CD16+ monocytes might favor better responses to therapeutic antibody treatment. Interestingly, a study by Romano *et al*. showed that melanoma patients who responded to treatment with ipilimumab had a significantly higher proportion of CD16+ monocytes as compared with non-responding patients^36^. Another study showed that CD16+ myeloid cells infiltration into the tumour mass in colorectal cancer patients represents a strong, novel and independent prognostic prosurvival factor^8^. Further studies are required to determine how the preferential expansion of this subset influences the progress of the different diseases. Moreover, we have shown the potential for human CD16+ monocytes to be effective mediators of ADCC against a range of cell types *in vitro* and therefore exploration of ways to exploit this potential *in vivo* could prove valuable in the clinical setting.

## Materials and Methods

### Isolation of effector cells from healthy donors and B-CLL patients

Blood samples of healthy donors from the blood bank and in-housed volunteers were approved by the NHG Domain Specific Review Board Singapore (Reference codes: 10–250 and 09–256 respectively) and blood and bone marrow samples from patients were approved by Singhealth Centralised Institutional Review Board Singapore (Reference code: 2013/1038/F and 2008/060/F respectively). All blood and bone marrow samples and procedures were carried out in accordance to guidelines of the Health Science Authority of Singapore. Informed consent for all samples was given in accordance to the Declaration of Helsinki. Peripheral blood mononuclear cells (PBMCs) were obtained using Ficoll density centrifugation. NK cells were isolated from PBMCs of healthy donors using the NK cell isolation kit (Miltenyi Biotec) to purity consistently ≥97%. For monocyte subset isolation, PBMCs were depleted of granulocytes, NK, B and T cells with α-CD15, α-CD56, α-CD19 and α-CD3 conjugated microbeads by magnetic-automated cell sorting (MACS) (Miltenyi Biotec). Monocyte subsets were identified from the depleted fraction using α-CD14 (clone 61D3, Ebioscience), α-CD16 (clone 3G8, BioLegend), α-NKp46 (clone 9E2, Miltenyi Biotec) and α-SLAN (clone DD.1, Miltenyi Biotec) and purified by FACS sorting according to the relative expression of CD14 and CD16 within the NKp46-negative population. The gating strategy for 2 monocyte subsets was CD14+CD16− (CD16−) and CD14+CD16+ (CD16+), and for 3 subsets was CD14++CD16− (classical), CD14++CD16+ (intermediate) and CD14+CD16++ (non classical). SLAN+ and SLAN− monocytes were sorted according to the expression of SLAN from within the non-classical subset gate. PBMCs obtained from whole blood of B-CLL patients and age matched healthy donors were depleted of T cells with α-CD3 conjugated microbeads by magnetic-automated cell sorting (MACS) (Miltenyi Biotec). NK cells and monocyte subsets were then identified from the depleted fraction using α-CD56 (clone NCAM16.2, BD Bioscience), α-CD14 and α-CD16 and purified by FACS sorting CD56+ cells for NK population and relative expression of CD14 and CD16 within the CD56−negative population for monocyte subsets. The resulting purity of each sorted subset was consistently ≥98%.

### Cell lines

All cell lines originated from ATCC, except HepG2.2.15, which was developed in one of our laboratories. A549 (human lung adenocarcinoma epithelial cell line) was maintained in complete IMDM (IMDM + 5% human serum (HS) + 1X penicillin-streptomycin (P/S). Raji (Burkitt lymphoma) and HepG2 (hepatocellular carcinoma) were maintained in complete RPMI (RPMI + 10% fetal calf serum (FCS) + 1X P/S) and SKBR3 (human breast adenocarcinoma cell line) was maintained in complete McCoy5A (McCoy5A + 10% FCS + 1X P/S). HepG2.2.15 was maintained in DMEM (high glucose) supplemented with 10% FCS, 1X P/S, 1 mM sodium pyruvate, 1X non-essential amino acids and 150 μg/ml G418. Only cell cultures with >95% viability were used in the experiments.

### Isolation of primary B cancer cells from leukemia patients

Bone marrow and peripheral blood from B-CLL patients obtained from the Singapore General Hospital Tissue Repository were purified using the human B-CLL cell isolation kit (Miltenyi Biotec). The purity was consistently 99% as measured by flow cytometry using α-CD20 (Clone 2H7, eBioscience).

### Monoclonal antibodies

The chimeric α-ganglioside GM2 monoclonal antibody (KM966) was a kind gift from Kyowa Hakko Kirin Co., Ltd. The chimeric α-CD20 (rituximab) and the humanized α-HER2 (trastuzumab) were purchased from Roche Pharmaceuticals. The TCR-like antibodies were generated in laboratory of Antonio Bertoletti. α-CD64 (clone 10.1, BioLegend), α-CD32 (clone AT10, Abcam), α-CD32a (clone IV.3, StemCell Technologies), α-CD32b (polyclonal, Abcam), α-CD16 (clone DJ130c, AbD Serotec), α-CD18 (Clone TS1/18, Biolegend), α-CD11a (Clone TS1/22, Thermo Scientific), α-CD11b (Clone CBRM1/5, eBioscience), α-CD11c (Clone 3.9, Biolegend), α-CD29 (Clone P5D2, R&D systems), α-TNFα (Clone Mab11, Biolegend), α-TNFR (Clone mab625, R&D systems) and an isotype-matched control antibody (MOPC21, BioLegend) were used for blocking experiments.

### Flow cytometry

To assess HER-2 and CD20 surface expression on SKBR3 and Raji cell lines respectively, cells were labeled with appropriate fluorochrome-conjugated antibodies for 30 mins at 4 °C in MACS buffer (1X PBS + 0.5% BSA + 2 mM EDTA). A549 was first labeled with unconjugated primary GM2 antibody before further labeling with fluorochrome-conjugated secondary antibodies. Fluorochrome-conjugated α-TNFR1 (Clone 16803, R&D systems) and α-CD16 (clone 3G8) were used for assessing the surface expression of TNFR and CD16 using protocol described for HER-2 and CD20.

### ADCC assay

#### DELFIA EuTDA-based cytotoxicity assay

ADCC for A549, SKBR3, HepG2 and primary B leukemic cell models was assessed using the DELFIA EuTDA-based cytotoxicity assay according to manufacturer’s instruction (Perkin Elmer). Approximately 2 × 10^6^ tumour cells/ml (i.e. target cells) were incubated with 5 μl of Bis(acetoxymethyl)-2-2:6,2 terpyridine 6,6 dicarboxylate (BATDA). Afterwhich, they were either untreated or coated with 10 μg/ml of the respective antibodies. 100 μl of effector cells (CD16+ monocytes, CD16− monocytes or NK cells) and 100 μl of target cells at the various effector to target (E:T) ratios with the target cells fixed at 1 × 10^5 ^cells/ml were co-incubated in 96-well U-bottomed plate. After 4 hours, 20 μl of supernatant was harvested from each well, incubated with 180 μl of Europium (Eu^3+^) solution in black-wall 96-well flat bottom plates and then read in a time-resolved fluorometer (Envision, Perkin Elmer). BATDA-labeled target cells alone with or without therapeutic antibodies were cultured in parallel to assess spontaneous lysis and in the presence of 2% Triton-X to measure maximum lysis. Percent specific lysis was defined as: (Sample lysis (counts) − Spontaneous lysis (counts))/(Maximum lysis (counts) − Spontaneous lysis (counts)) × 100%.

#### Radioactivity-based cytotoxicity assay

Raji B lymphoma (i.e. target cells) were incubated with 60–200 μCi Sodium Chromate (Na_2_^51^CrO_4_) (Perkin Elmer) for 3 hrs at 37 °C before co-culture with appropriate numbers of effector cells dependent on the E:T ratios. After 4 hrs, 100 μl supernatant was harvested for quantification using a γ-counter. Radiolabelled target cells alone, with or without therapeutic antibodies were cultured in parallel to measure spontaneous lysis, and in the presence of 2% Triton-X to measure maximum lysis. Percent specific lysis was calculated as described above.

### ADCC upon antibody cross-linking

Either BATDA-labeled SKBR3 or primary B leukemic cells were left untreated or coated with 10 μg/ml of the respective therapeutic antibodies. Afterwhich, they were pre-incubated in the presence or absence of 10 μg/ml of α-IgG, Fcγ fragment specific (Jackson Immunoresearch) for 30 mins at 37 °C prior to the addition of various concentrations of recombinant human (rh)TNFα (Peprotech) and incubated for 4 hours at 37 °C.

### TNFα ELISA

Uncoated or rituximab-coated Raji cells were fixed in 1% paraformaldehyde for 20 mins before co-culturing with CD16+ monocytes for 4 hours at an E:T ratio of 10:1. Afterwhich, supernatants were collected and the secreted TNFα was assessed using the human TNFα DuoSet ELISA according to manufacturer’s instructions (R&D systems).

### “Innocent bystander” ADCC assay

2 × 10^5^ CD16+ monocytes were incubated with a mixture of 1 × 10^4^ each of SKBR3 cells with or without trastuzumab. In one set, only the SKBR3 cells coated with trastuzumab was labeled with BATDA, and in the second set, only the uncoated SKBR3 was labeled with BATDA.

### Live cell imaging

SKBR3 cells were grown on 8-well slides (IBIDI) in complete McCoy to 70–80% confluence. 10 μg/ml of trastuzumab in fresh media was added and incubated for 30 mins before the cells were washed to remove any unbound trastuzumab. Then 5 × 10^5 ^cells/ml of CD16+ monocytes were added. Real time cellular events were visualized using an FV-1000 confocal system with an inverted Olympus IX81 microscope. During imaging, cells were kept in a humidifier maintained at 37 °C and 5% CO_2_. Events were visualized at 200x magnification, and images were captured at 20 sec intervals for up to 2 hrs.

### Treatment of effector cells

Purified monocyte subsets and NK cells were pre-incubated at 37 °C for 5 hrs with 100 ng/ml LPS (*E. coli* 0111:B4; Sigma Aldrich), 10 μg/ml R848 (Invivogen), 100 ng/ml rhIL-12 (Immunotools), 100 ng/ml rhIL-15 (R&D Systems), 20 ng/ml rhIFN-γ (Immunotools), 8 μg/ml S100A9 (Origene Technologies) or 100 ng/ml HMGB1 (R&D Systems) before they were used for ADCC assay with BATDA-labeled GM2-coated A549 as target cells at an E:T ratio of 10:1. Freshly isolated CD16− monocytes were cultured in complete IMDM either in the absence or presence of 50 ng/ml rhM-CSF (Immunotools), 50 ng/ml rhTGF-β (R&D Systems) or 50 ng/ml rhIL-10 (Immunotools) at 37 °C for 24 hrs before their ADCC activity was assessed on trastuzumab-coated SKBR3 at a 10:1 E:T ratio.

### Nucleoporation of CD16− monocytes

The plasmid Myc-DDK-tagged ORF clone of human FCGR3A, transcript variant 1 (OriGene, USA) was propagated in XL10^®^-Gold competent cells (Stratagene) and subsequently purified using QIAfilter plasmid Midi kit (Qiagen). The plasmid was linearized and then CD16 mRNA was generated with the mMESSAGE mMACHINE T7 Ultra kit according to manufacturer’s instructions (Ambion).

5 × 10^6^ CD16− monocytes resuspended in 100 μl of Human Monocyte Nucleofactor Solution (Lonza) were mixed with either 20 μg of purified CD16 mRNA or without mRNA (mock) and electroporated in AMAXA-certified cuvettes using a Nucleofector apparatus (Lonza) using program Y-001. After electroporation, cells were immediately mixed with complete IMDM and transferred to 12-well plates containing the same medium. After 10 hrs, the cells were washed once and used for ADCC assay with BATDA-labeled trastuzumab-coated SKBR3 cells at an E:T ratio of 10:1.

### Statistical analysis

All data were analysed using Prism 6 (Graphpad software) and presented as mean ± SD. *P* values < 0.05 were considered to indicate statistically significant difference. Details of the tests performed to determine statistical significant difference are described in the figure legends.

## Additional Information

**How to cite this article**: Yeap, W. H. *et al*. CD16 is indispensable for antibody-dependent cellular cytotoxicity by human monocytes. *Sci. Rep.* **6**, 34310; doi: 10.1038/srep34310 (2016).

## Supplementary Material

Supplementary Information

Supplementary Movie 1
