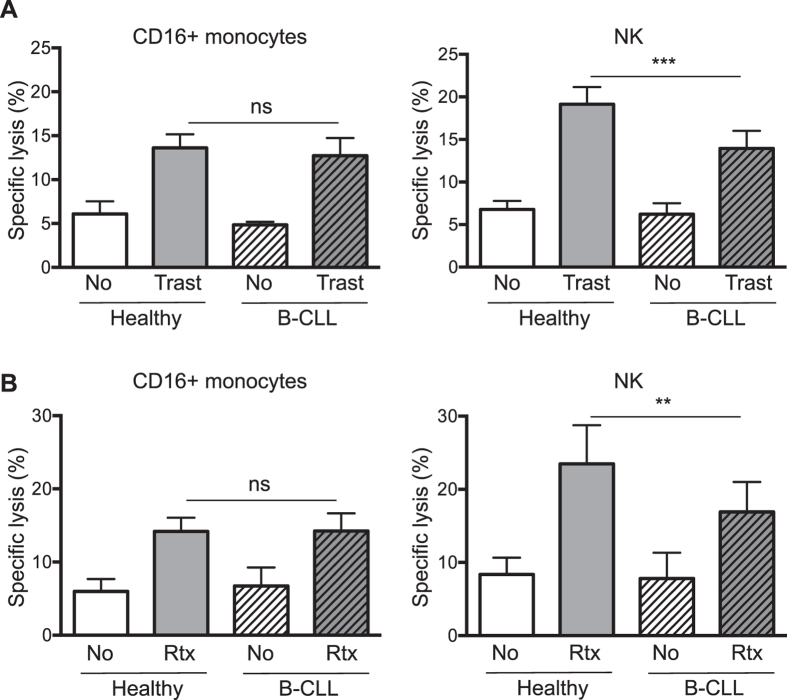# Corrigendum: CD16 is indispensable for antibody-dependent cellular cytotoxicity by human monocytes

**DOI:** 10.1038/srep46202

**Published:** 2017-04-07

**Authors:** Wei Hseun Yeap, Kok Loon Wong, Noriko Shimasaki, Esmeralda Chi Yuan Teo, Jeffrey Kim Siang Quek, Hao Xiang Yong, Colin Phipps Diong, Antonio Bertoletti, Yeh Ching Linn, Siew Cheng Wong

Scientific Reports 6: Article number: 34310; 10.1038/srep34310 published online 09 27 2016; updated: 04 07 2017.

This Article contains errors in Figure 3, which was inadvertently split in two, resulting in the omission of Figure 7 and the mislabelling of Figures 4, 5 and 6. The correct Figures 3, 4, 5, 6 and 7 appear below as Figures 1, 2, 3, 4 and 5 respectively. The Figure legends are correct.

## Figures and Tables

**Figure 1:**
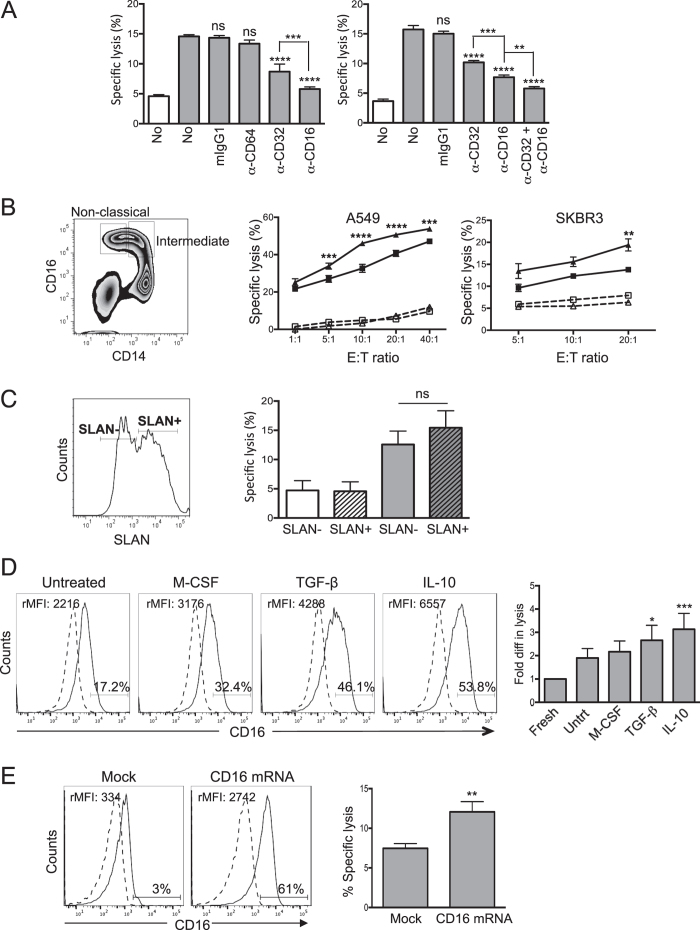


**Figure 2:**
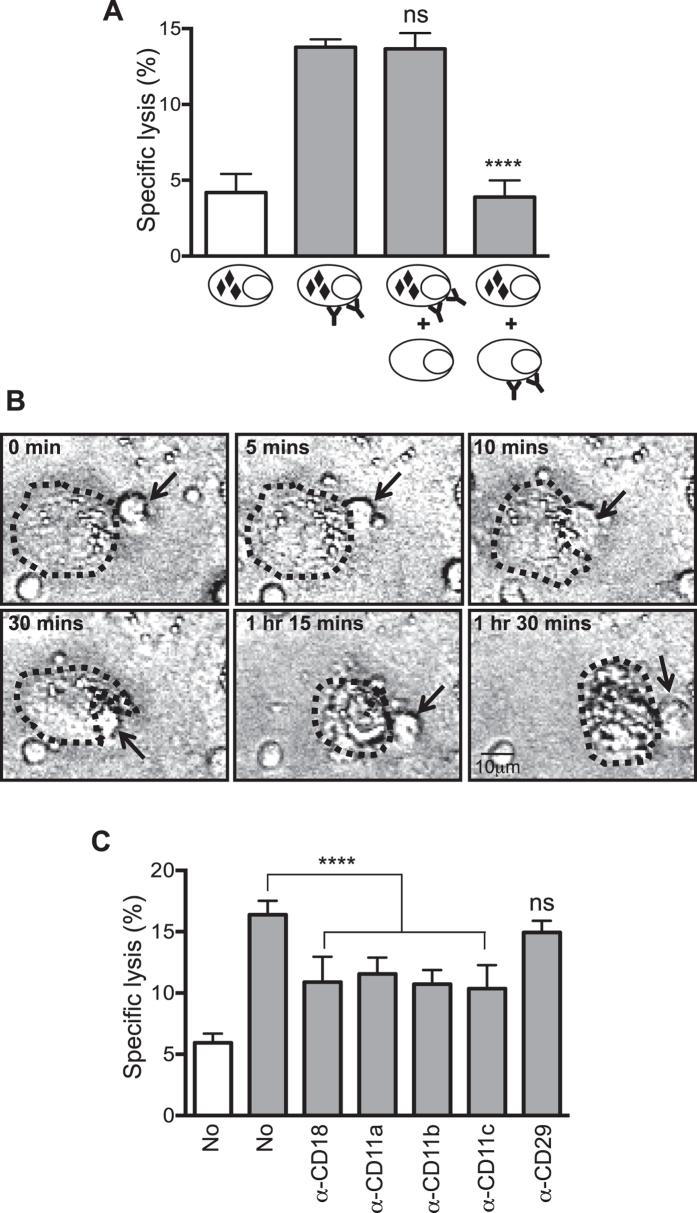


**Figure 3:**
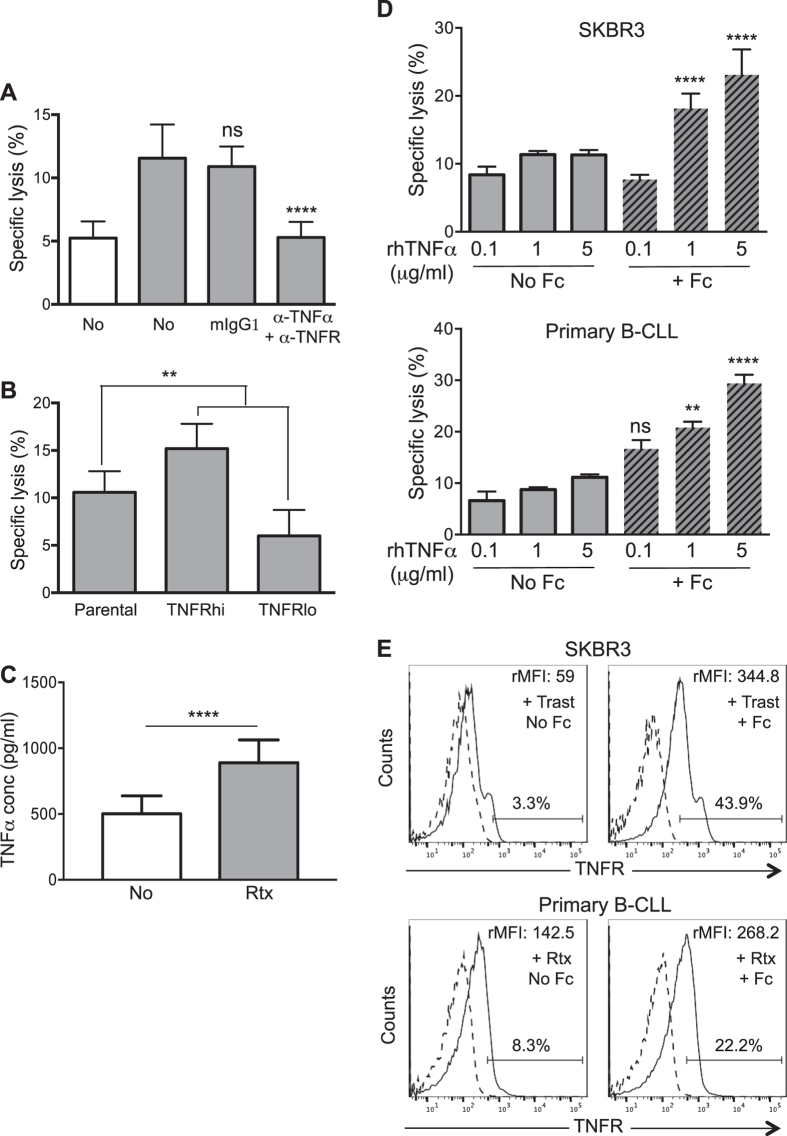


**Figure 4:**
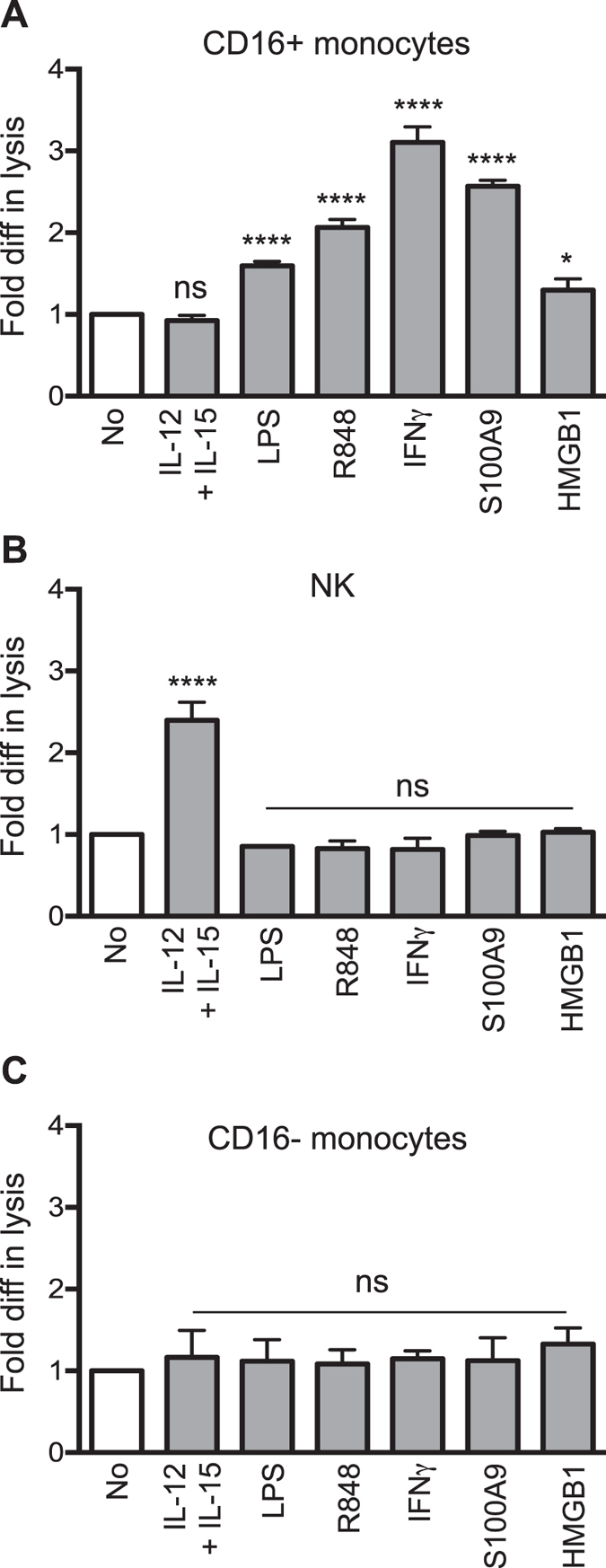


**Figure 5:**