# Supplementary material for: CD16 is indispensable for antibody-dependent cellular cytotoxicity by human monocytes
Source: Sci Rep. 2016 Sep 27;6:34310. doi: 10.1038/srep34310 (PMC5037471; doi:10.1038/srep34310)
Supplement: Supplementary Information [file srep34310-s1.pdf]

CD16 is indispensable for antibody-dependent cellular cytotoxicity by human monocytes.

Wei Hseun Yeap[1], Kok Loon Wong[1], Noriko Shimasaki[2], Esmeralda Chi Yuan Teo[3], Jeffrey Kim Siang Quek[3], Hao Xiang Yong[3], Colin Phipps Diong[3], Antonio Bertoletti[4,5], Yeh Ching Linn[3], Siew Cheng Wong[1*]

[1]Singapore Immunology Network (SIgN), Agency for Science, Technology and Research (ASTAR), 8A Biomedical Grove, #04-06, Immunos, Singapore 138648, Singapore.
[2]Department of Pediatrics, National University of Singapore, Centre for Translational Medicine, 14 Medical Drive Singapore 117599, Singapore. [3]Department of Haematology, Singapore General Hospital, Outram Road, Singapore 169608, Singapore.
[4]Singapore Institute for Clinical Sciences, Brenner Centre for Molecular Medicine, 30 Medical Drive, Singapore 117609, Singapore.
[5] Duke-NUS Medical School, 8 College Road, Singapore 169857, Singapore.

Supplementary Figure 1

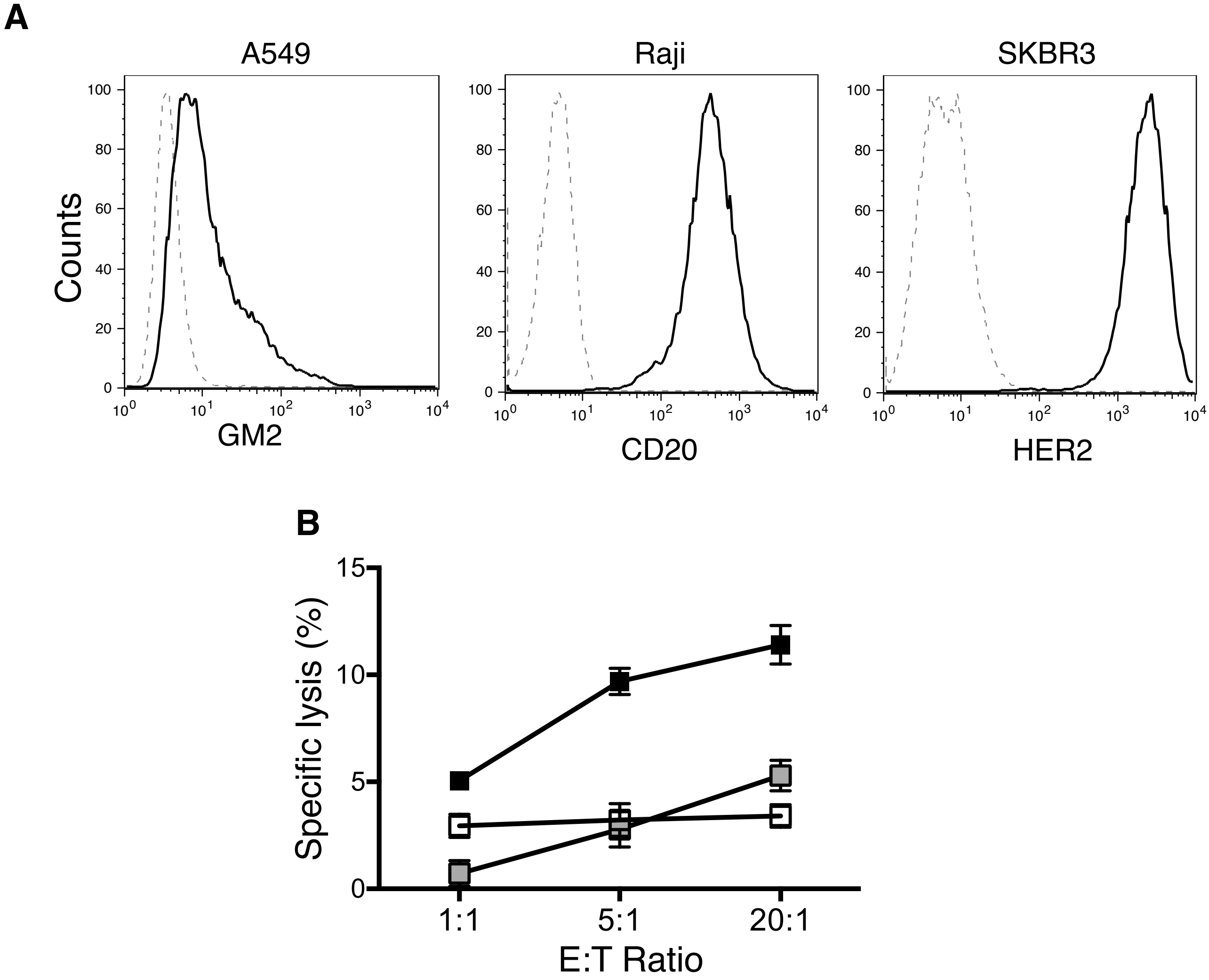

Supplementary Figure 2

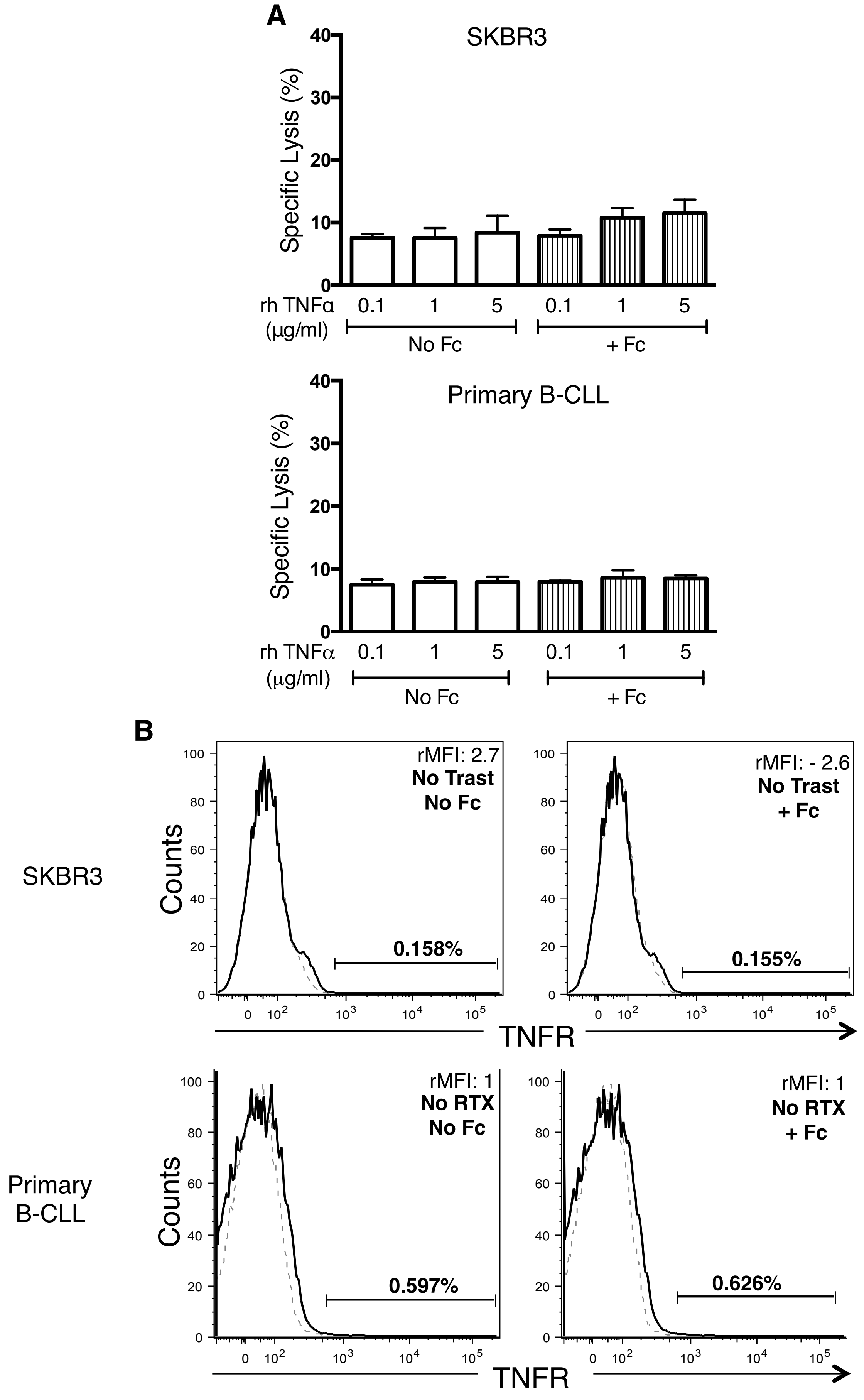

Supplementary Figure 3

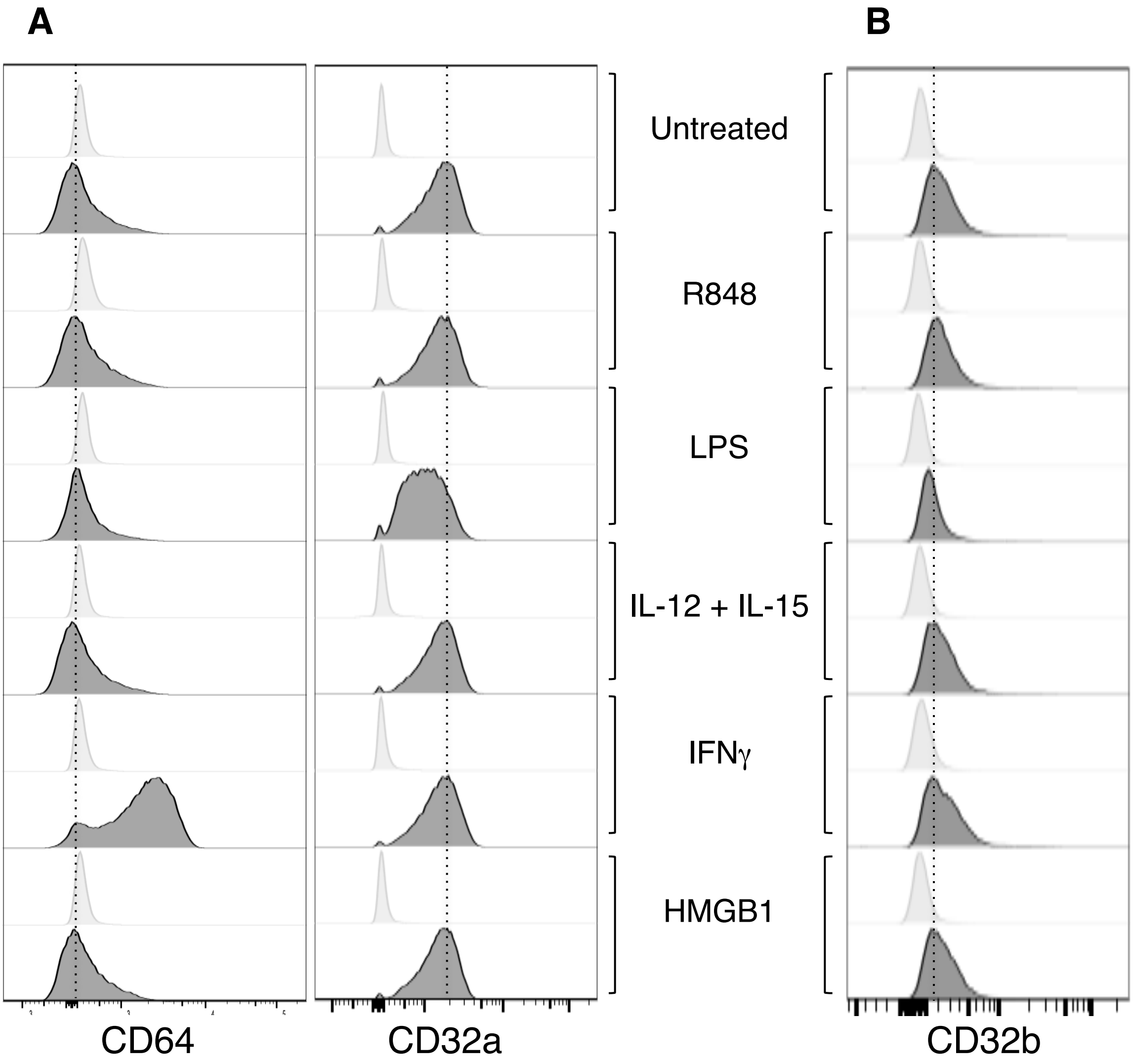

Figure Legend

Supplementary Figure 1: ADCC by CD16+ monocytes.

(A) Expression of surface antigens on cancer cell lines. The expression levels of GM2, CD20 and HER2 on A549 adenocarcinoma (left panel), Raji B lymphoma cells (middle panel) and SKBR3 breast adenocarcinoma (right panel) were measured by flow cytometry using KM966, rituximab and trastuzumab antibodies respectively. Specific labelling is indicated by solid lines while isotype-matched control antibodies were used to assess non-specific labeling (dotted grey line). **(B)** ADCC by CD16+ monocytes is antigen-specific. CD16+ monocytes were co-cultured with A549 cells either uncoated (grey squares) or pre-coated with either KM966 (black squares) or Rituximab (irrelevant ab; white squares) at the indicated E:T ratios. Data shown is a representative of 3 independent experiments. Data plotted are mean ± SD of triplicate wells.

Supplementary Figure 2: Susceptibility of target cells to TNFα mediated lysis.

(A) Uncoated SKBR3 (top panel) and primary B leukemic cells (bottom panel) were treated with the indicated concentrations of recombinant human TNFα (rhTNFα) in the absence (No Fc) or presence (+ Fc) of anti human IgG. Data shown are representative of 2 independent experiments for SKBR3 and 3 independent experiments for primary B leukemic cells and plotted as specific lysis with respect to that of untreated cells. **(B)** Uncoated SKBR3 (top histograms) and primary B leukemic cells (bottom histograms) were treated with 5 mg/ml rhTNFα in the absence (No Fc) or presence (+ Fc) of anti human IgG. Histogram plots of TNFR expression labelled with TNFR antibody (black solid line) versus background labelling with isotype-matched control antibody (grey dashed line). Percentages indicate the proportion of positively stained cells. Data shown are representative of 1 independent experiment for SKBR3 and 3 independent experiments for primary B leukemic cells.

Supplementary Figure 3: Expression of CD64 and CD32 isoforms on treated CD16+ monocytes.

Histogram plots showing **(A)** surface CD64 and CD32a expression or **(B)** intracellular CD32b expression on CD16+ monocytes that were either left untreated or treated with the various indicated stimuli for 5 hours. Specific labelling is indicated by black lines while isotype-matched control antibodies were used to assess non-specific labeling (grey lines). The dotted lines indicate the expression of the respective molecules of the untreated samples. Data shown are representative of 2 independent experiments.

Supplementary Movie: Time-lapse imaging of ADCC by a CD16+ monocyte on a trastuzumab-coated SKBR3 cell.
